# Investigation on the Lung Function of General Population in Ilam, West of Iran, as a City Exposed to Dust Storm

**DOI:** 10.5539/gjhs.v7n3p298

**Published:** 2015-01-14

**Authors:** Ali Amarloei, Ahmad Jonidi Jafari, Hassan Asilian Mahabadi, Kheirollah Asadollahi, Heshmatollah Nourmoradi

**Affiliations:** 1 Department of Environmental Health Engineering, School of Medical Sciences, Tarbiat Modares University, Tehran, Iran; 2 Department of Environmental Health Engineering, School of Public Health, Iran University of Medical sciences, Tehran, Iran; 3 Department of Occupational Health, School of Medical Sciences, Tarbiat Modares University, Tehran, Iran; 4 Department of Epidemiology and Statistics, School of Medicine, Ilam University of Medical Sciences, Ilam, Iran; 5 Department of Environmental Health Engineering, School of health, Ilam University of Medical Sciences, Ilam, Iran

**Keywords:** Iran, Ilam, air pollution, dust storm, lung function

## Abstract

**Background:**

Dust storm is one of the most important natural sources of air pollution in the Middle East that has caused a major concern in recent years. The aim of this study was to evaluate the respiratory tract function of people living in Ilam city (Iran) during dust storm.

**Methods:**

A sample size of 250 people was selected and the cluster sampling was randomly used from 13 health centers in Ilam city. Pulmonary function test (PFT) was determined via a standard spirometry apparatus. Vital capacity (VC), Forced Vital capacity (FVC), FVC in first second (FEV_1_), FEV_1_/VC, FEV_1_/FVC, peek expiratory flow (PEF), forced expiratory flow (FEF_25-75%_), forced expiratory flow (FEF_25–75%_), forced expiratory flow (FEF_75–85%_), forced mid flow time (FMFT) and maximum voluntary ventilation (MVV) were measured.

**Results:**

Mean values of respiratory capacities measured in all participants excluding FEV_1_/VC and FMFT were less than predicted mean values by ECCS reference. 21.6% of the population suffered from obstructive lesions. This value among males (24.1%) was more than females (19.6%). This could be related to more exposure (outdoor jobs) of males with dust storms.

**Conclusion:**

The results also showed a negative significant relationship between duration of inhabitance in Ilam city and all respiratory capacities. Further studies are needed for confident confirmation of whether reduction of respiratory capacities among Ilamian people is only related to dust storms.

## 1. Introduction

In recent decades, air pollution in many parts of the world has caused increasing concerns about adverse effects on human health (Teather et al., 2013). According to the World Health Organization (WHO), about 1.4% of the total mortalities in the world have been allocated to air pollution (Evans et al., 2013). Although human activities such as industries and transportation have mainly caused air pollution, but natural sources can also play substantial role in the creating air pollution (Teather et al., 2013). Dust storm is one of the most important natural sources of air pollution that has introduced 800 trillion grams of particulate matter (PM) in Asia annually (Shahsavani et al., 2012).

The health effects of dust storms especially in the Middle East are a major concern in recent years (Teather et al., 2013; Shahsavani et al., 2012; Chan and Ng, 2011). PM_10_ and PM_2.5_ (particle matter with diameter equal or less than 10 and 2.5 μm, respectively) are the important constituents of dust storm (Li et al., 2010; Meng and Zhang, 2007). An increase of the airborne particles concentration (PM_10_ and PM_2.5_) over dust storm phenomenon, due to their deep penetration into the lower respiratory tract (alveoli), has resulted in a range of illnesses and deaths (Geng et al., 2006; Chen et al., 2004; Meng and Zhang, 2007; Bourotte et al., 2007). It is commonly believed that PM_2.5_ creates the most dangerous inability to lung performance (Teather et al., 2013). Most current studies reported that 3.5 million cardiovascular and 220,000 respiratory tract cancer deaths due to PM_2.5_ are being annually assigned (Evans et al., 2013). Epidemiological studies also indicated that ischemic heart diseases, respiratory diseases such as asthma, rhinitis and pneumonia, lung dysfunction, daily mortality and hospital admissions were increased during 2 days after dust events (Geng et al., 2006; Kang et al., 2012; Hong et al., 2010; Chien et al., 2012; Meng and Zhang, 2007; Teather et al., 2013; Wu et al., 2010; Pal et al., 2010). Cheng et al. 2012 reported a significant relation between Asian Dust Storm (ADS) episodes and daily morbidity because of pneumonia during one day after the event (Kang et al., 2012). Many researchers have also shown the significant increase in hospital admission due to pneumonia, ischemic heart diseases, and other heart diseases within dust events (Chien et al., 2012).

Lung function is considered as one of the major markers of safety for respiratory and cardiac systems (Downs et al., 2007). Lung performance can be affected by air pollution, especially by suspended particles (Rinne et al., 2006; Moini et al., 2010). Several studies have been conducted on the relation between suspended particles exposure and lung function in young and elderly people (Mölter et al., 2013; Curjuric et al., 2010). The improvement in air quality was resulted in diminishing cardiopulmonary deaths and an enhancement of lung function in children (Imboden et al., 2009). Schwartz et al. (2009) indicated that the dwellers of more air polluted regions have worse lung functions (Imboden et al., 2009). They also reported that long term exposure to air particulate matter decreases the lung function (Imboden et al., 2009).

The west (such as Ilam city) and southern west of Iran appear to be influenced by dust carried by the Shamal wind, a hot northwest wind that is dominant during the spring and carries large quantities of dust from Iraq deserts (Middleton and Goudie, 2006). In recent decade, Ilam city has been generally experienced dust events originating from the above mentioned source. Despite of its importance, this phenomenon has not been studied so far. To the best of our knowledge, there is no published document about the status of the lung function among general population in Ilam city. Therefore, the main objective of this study was to evaluate the respiratory tract function of people living in this region during dust storms.

## 2. Methods

This study was conducted from August to September 2013 in Ilam city with population of 172000 people. Including criteria in this study were: 18 years old persons and more with at least 5 years inhabitance in Ilam city, no hereditary and familial history of respiratory diseases, no history of addiction and smoking, no direct contact with job inhaling pollutants, no history of acute respiratory infection in last two weeks and no usage of bronchodilators (Golshan and Nematbakhsh, 2000). A sample size of 250 people was selected based upon the average and standard deviation values of previous studies (Moini et al., 2010). Then, the cluster sampling was randomly used through 13 health centers located in Ilam city. A standard spirometry questionnaire including demographic characteristics (age, height, gender, marital status, education, job, residence duration in Ilam city), respiration status and other physical symptoms and problems during days with and without dust storms, past medical history, familial history of respiratory diseases, drug usage and smoking for each participant was completed (Jafari & Assari, 2004). Stand height and weight without wearing shoes was measured by using calibrated scale and stadiometer.

Pulmonary function test (PFT) was determined via a Vitalograph spirometry apparatus (Compact II, England) (Society and Physicians, 2003; He et al., 2010; Ahmadial et al., 2006; Moini et al., 2010; Pal et al., 2010). According to the American thoracic society (ATS) standards, at least 3 acceptable tests were performed for each individual. The device was calibrated once for every three individuals by 1-liter syringe. Vital capacity (VC), Forced Vital capacity (FVC), Forced expiratory volume in first second (FEV_1_), FEV_1_/VC, FEV_1_/FVC, peek expiratory flow (PEF), forced expiratory flow at 25%, 50%, 75% of volume as a percentage of FVC (FEF_25%_, FEF_50%_, FEF_75%_), forced expiratory flow from 25–75% of the FVC (FEF_25–75%_), forced expiratory flow from 75–85% of FVC (FEF_75–85%_), forced mid flow time (FMFT) and maximum voluntary ventilation (MVV) parameters were measured. Then, the information achieved from the questionnaires and spirometry results were extracted and analyzed by SPSS version 16. A p-value of <0.05 was considered significant. In this research, the European community for coal and steel (ECCS) reference was used to evaluate the respiratory status of studied population (Alizadeh et al., 2006; Boskabady et al., 2002). T-test and Chi-square test were applied to compare mean pulmonary capacities data with reference scales (ECCS) and to compare the prevalence data of respiratory symptoms in dusty days with normal days, respectively.

## 3. Results

The anthropometric parameters values of the participants are shown in Table 1. Of the 250 participants, 44.8% and 55.2% were male and female, respectively. Mean age of total participants was 38.23±12.35 years. The minimum and maximum age of participants was 18 and 90 years, respectively. Mean habitation time (year) of the participants in Ilam city was 32.70±14.61 years.

**Table 1:** Demographic and anthropometric characteristics of the participants.

Parameter	Males (n=112)		Females (n=138)		Total (n=250)	
		
	Mean	SD	Mean	SD	Mean	SD
Age (years)	38.10	13.09	38.33	11.76	38.23	12.35
Height (cm)	172.37	8.57	160.49	7.20	165.81	9.81
Weight (kg)	76.99	13.13	67.67	11.63	71.85	13.15
BMI (kg/m^2^)	25.85	3.52	26.32	4.51	26.11	4.09
Habitation (year)	33.00	14.46	32.46	14.78	32.70	14.61

The results of the pulmonary tests are indicated in Table 2. All the studied parameters were significantly different in males and females (p<0.05). Lung function parameters were compared with predicted values of ECCS reference. There were significant differences between all the parameters except for FVC and PEF in total participants, FVC, FEV_1_/VC, PEF and MVV in males and FVC in females (P<0.05). These values were located within 61.25%–128.95% of those derived from prediction equations values for ECCS of total subjects. The largest variations were for FMFT which was significantly higher (128.95%) and FEF_75%_ which was significantly lower (61.25%) in males compared with the reference values. All the spirometric values (VC, FVC, FEV1, PEF, FEF_25%_, FEF_50%_, FEF_75%_, FEF_25- 75%_, FEF_75-85%_, FMFT and MVV) in males were greater than of females.

**Table 2:** Mean lung volumes and capacities in healthy adult Ilamian males and females compared with ECCS reference values.

	Males (n = 112)				Females (n = 138)				Total (n=250)			

	Measured values (Mean±SD)	ECCS values (Mean±SD)	% difference^#^	p-value	Measured values (Mean±SD)	ECCS values (Mean±SD)	% difference^#^	p-value	Measured values (Mean±SD)	ECCS values (Mean±SD)	% difference^#^	p-value
VC (l)	4.32±0.89	4.66±0.88	92.70	0.000	3.14±0.67	3.31±0.56	94.86	0.000	3.67±0.97	3.92±0.98	93.65	0.000
FVC (l)	4.41±0.90	4.47±0.81	98.66	0.196	3.30±0.65	3.25±0.54	101.54	0.159	3.80±0.94	3.80±0.91	100.02	0.974
FEV_1_ (l)	3.49±0.75	3.73±0.68	93.57	0.000	2.66±0.55	2.80±0.49	95.00	0.000	3.03±0.77	3.22±0.74	94.24	0.000
FEV_1_/VC	81.09±8.00	80.54±2.39	100.68	0.450	84.53±8.15	81.73±2.24	103.43	0.000	82.99±8.25	81.2±2.38	102.20	0.000
FEV1/FVC	79.18±5.60	83.46±2.32	94.87	0.000	80.64±6.30	85.93±1.68	93.84	0.000	79.98±6.03	84.82±2.34	94.29	0.000
PEF (l/sec)	535.87±144.35	528.19±75.92	101.45	0.491	377.79±93.16	397.66±44.82	95.00	0.002	448.61±142.4	456.14±88.91	98.35	0.222
FEF_25%_(l/sec)	7.10±1.85	7.61±1.00	93.30	0.001	5.55±1.39	5.87±0.56	94.55	0.002	6.24±1.78	6.65±1.18	93.83	0.000
FEF_50%_ (l/sec)	3.99±1.30	4.93±0.63	80.93	0.000	3.39±1.00	4.15±0.41	81.69	0.000	3.66±1.18	4.50±0.65	81.33	0.000
FEF_25-75%_(l/sec)	3.37±1.18	4.31±0.67	78.19	0.000	2.79±0.91	3.64±0.91	76.65	0.000	3.05±1.07	3.94±0.65	77.43	0.000
FEF_75%_(l/sec)	1.35±0.60	2.16±0.44	62.5	0.000	1.11±0.50	1.85±0.33	60.00	0.000	1.22±0.56	1.99±0.41	61.25	0.000
FEF_75-85%_(l/sec)	0.88±0.48	1.20±0.31	73.33	0.000	0.73±0.40	1.09±0.27	66.97	0.000	0.80±0.44	1.14±0.30	70.00	0.000
FMFT (sec)	0.71±0.22	0.59±0.05	120.34	0.000	0.65±0.25	0.48±0.03	135.42	0.000	0.68±0.24	0.53±0.07	128.95	0.000
MVV (l/min)	130.79±28.11	133.83±19.99	97.73	0.052	99.91±20.63	104.79±12.67	95.34	0.000	113.74±28.69	117.80±21.81	96.55	0.000

VC: Vital capacity; FVC: forced vital capacity; FEV_1_: forced expiratory volume in one second; PEF: peak expiratory flow; FEF_25–75_: expiratory flow from 25–75% of the vital capacity; FEF_25_, FEF_50_, FEF_75_: instantaneous expiratory flows at 25%, 50% and 75% of FVC, respectively; FEF_75–85_: expiratory flow from 75–85% of the vital capacity; FMFT: Forced mid flow time; MVV: maximum voluntary ventilation. SD = standard deviation; n = total number of participants.

# Percentage difference between the measured values with reference values.

The spirometric values also showed a moderate to strong positive correlation with height (Table 3). But, these correlation values were less severe in females. The correlation between spirometric values and weight was weaker but still significant for certain parameters. As it was also expected, there was an inverse correlation between age and spirometric values. The correlation between spirometric values and BMI (body mass index) was not significant in most cases (p<0.05). However, this correlation had inverse relation among females and for certain parameters were significant. There was also a significant inverse correlation between spirometric values and duration of habitation in Ilam city (p<0.05).

**Table 3:** Pearson’s correlation coefficients between spirometric parameters in adult residents in Ilam according to different variables

Parameter	Females (n = 138)					Males (n = 112)				

	Age (year)	Height (cm)	Weight (kg)	BMI (kg/m^2^)	Habitation (year)	Age (year)	Height (cm)	Weight (kg)	BMI (kg/m^2^)	Habitation (year)
VC (l)	-0.419**	0.718**	0.417**	0.006	-0.355**	-0.455**	0.558**	0.071	-0.233**	-0.196*
FVC (l)	-0.458**	0.734**	0.423**	0.003	-0.408**	-0.516**	0.629**	0.084	-0.257**	-0.279**
FEV_1_ (l)	-0.508**	0.712**	0.415**	0.004	-0.427**	-0.631**	0.570**	0.034	-0.277**	-0.370**
FEV_1_/VC	-0.245**	0.005	-0.009	-0.025	-0.207*	-0.350**	-0.067	-0.060	-0.026	-0.256**
FEV1/FVC	-0.261**	0.030	0.001	-0.035	-0.143	-0.426**	-0.071	-0.117	-0.087	-0.302**
PEF (l/sec)	-0.241*	0.477**	0.362**	0.118	-0.217*	-0.506**	0.492**	0.187*	-0.076	-0.276**
FEF_25%_(l/sec)	-0.264**	0.448**	0.345**	0.109	-0.232*	-0.514**	0.406**	0.171*	-0.046	-0.290**
FEF_50%_ (l/sec)	-0.387**	0.452**	0.307**	0.049	-0.305**	-0.487**	0.270**	0.024	-0.123	-0.322**
FEF_25-75%_(l/sec)	-0.435**	0.462**	0.292**	0.021	-0.334**	-0.558**	0.291**	0.001	-0.159	-0.354**
FEF_75%_(l/sec)	-0.517**	0.434**	0.172	-0.105	-0.405**	-0.550**	0.237**	-0.127	-0.258**	-0.355**
FEF_75-85%_(l/sec)	-0.485**	0.387**	0.110	-0.150	-0.354**	-0.544**	0.205*	-0.193*	-0.310**	-0.368**
FMFT (sec)	0.266**	-0.033	-0.060	-0.036	0.148	0.413**	0.086	0.000	-0.040	0.270**
MVV (l/min)	-0.507**	0.711**	0.415**	0.005	-0.425**	-0.632**	0.569**	0.033	-0.277**	-0.371**

VC: Vital capacity; FVC: forced vital capacity; FEV_1_: forced expiratory volume in one second; PEF: peak expiratory flow; FEF_25–75_: expiratory flow from 25–75% of the vital capacity; FEF_25_, FEF_50_, FEF_75_: instantaneous expiratory flows at 25%, 50% and 75% of FVC, respectively; FEF_75–85_: expiratory flow from 75–85% of the vital capacity; FMFT: Forced mid flow time; MVV: maximum voluntary ventilation.

**. Correlation is significant at the 0.01 level (2-tailed).

*. Correlation is significant at the 0.05 level (2-tailed).

The frequency of respiratory complications in Ilam city inhabitants based on spirometric tests is shown in Table 4. The obstructive pulmonary complication is the most common problem in participants. This complication in males and females was 24.1% and 19.6%, respectively. The frequency of respiratory symptoms in dust storms and normal days according to gender is shown in Table 5. These complications during dust storms were significantly higher than normal days (p<0.05).

**Table 4:** The frequency of respiratory complications in Ilam city inhabitants.

Phenomenon	Males (n = 112)		Females (n = 138)		Total (n=250)	
		
	Frequency	%	Frequency	%	Frequency	%
Normal	83	74.1	105	76.1	188	75.2
Obstruction	27	24.1	27	19.6	54	21.6
Restriction	2	1.8	4	2.9	6	2.4
Mixed	0.0	0.0	2	1.4	2	0.8

**Table 5:** Comparison of the frequency of respiratory symptoms during dust storm and non-dust storm days

Symptoms	Males (n = 112)						Females (n = 138)						Total (n=250)					
		
	Dust storm days		Normal day		OR	P-value	Dust storm days		Normal day		OR	P-value	Dust storm days		Normal day		OR	P-value
		
	Frequency	%	Frequency	%			Frequency	%	Frequency	%			Frequency	%	Frequency	%		
Cough	53	47.3	15	13.4	3.5	0.000	79	57.2	14	10.1	5.6	0.000	132	52.8	29	11.6	4.7	0.000
Dyspnea	67	59.8	13	11.6	5.2	0.001	87	63	21	15.2	4.2	0.000	154	61.6	34	13.6	4.5	0.000

* P-value< 0.05.

**Figure 1:**
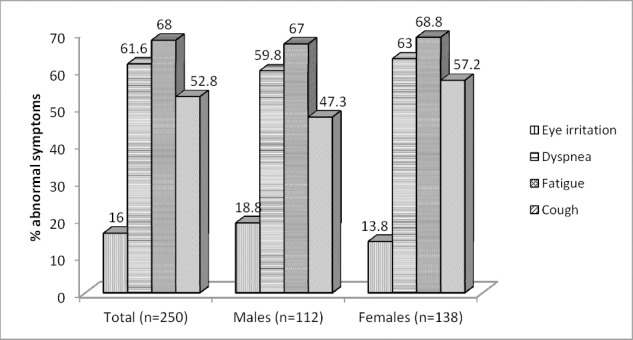
The frequency of abnormal symptoms during dust storm days

The frequencies of abnormal symptoms during dust storm days are indicated by Figure 1. The most common physical symptoms reported by participants during dust storms were fatigue, dyspnea and cough.

**Table 6:** Comparison of lung volume values obtained in the present study with other studies

Study	FVC(lit)			FEV_1_(lit)			FEV_1_/FVC			PEF(l/s)			FEF_25%_(l/s)			FEF_50%_(l/s)			FEF_25-75%_(l/s)			FEF_75%_(l/s)		
	Total	Male	Female	Total	Male	Female	Total	Male	Female	Total	Male	Female	Total	Male	Female	Total	Male	Female	Total	Male	Female	Total	Male	Female
Present study	3.80	4.41	3.30	3.22	3.49	2.66	79.98	79.18	80.64	7.48	8.93	6.30	6.24	7.10	5.55	3.66	3.99	3.39	3.05	3.37	2.79	1.22	1.35	**1.11**
(Golshan et al., 2007)	-	4.48	3.27	-	3.88	2.88	-	86.61	88.1	-	7.49	5.59	-	7.22	5.27	-	5.1	3.88	-	4.35	3.36	-	1.98	**1.65**
(Amra et al., 2006)	-	4.52	3.33	-	3.9	2.92	-	86.41	88.18	-	7.54	5.61	-	-	-	-	-	-	-	-		-	-	-
(Golshan et al., 2003)	-	4.68	3.17	-	4.05	2.78	-	86.76	88.02	-	10.58	6.77	-	10.58	6.77	-	6.02	4.6	-	4.94	3.74	-	2.25	**1.71**
(Razi et al., 2005)	-	4.47	3.03	-	3.94	2.72	-	-	-	-	-	-	-	-	-	-	-	-	-	4.92	3.59	-	-	-
(Boskabady et al., 2002)	-	4.4	3.13	-	3.78	2.77	-	-	-	-	8.99	6.12	-	7.98	5.68	-	4.96	3.93	-	4.39	3.54	-	2.28	**1.96**
(Sharifian et al., 2007)	3.60	4.33	3.0	3.12	3.74	2.61	-	-	-	5.95	7.6	4.5	-	-	-	-	-	-	3.68	4.45	3.04	-	-	-
(Alizadeh et al., 2006)	3.9	4.58	3.27	3.54	4.12	2.99	90.74	90.22	91.23	-	-	-	-	-	-	-	-	-	4.54	5.16	3.95	-	-	-
(Etemadinezhad and Alizadeh, 2011)	3.94	4.59	3.22	3.38	3.9	2.8	-	-	-	-	-	-	-	-	-	-	-	-	3.98	4.46	3.43	-	-	-
(Moini et al., 2010)	3.84	-	-	3.25	-	-	84.68	-	-	6.74	-	-	-	-	-	-	-	-	3.69	-	-	-		-
(Moini et al., 2010)	3.69	-	-	3.17	-	-	85.60	-	-	6.48	-	-	-	-	-	-	-	-	3.65	-	-	-		-

VC: Vital capacity; FVC: forced vital capacity; FEV_1_: forced expiratory volume in one second; PEF: peak expiratory flow; FEF_25–75_: expiratory flow from 25–75% of the vital capacity; FEF_25_, FEF_50_, FEF_75_: instantaneous expiratory flows at 25%, 50% and 75% of FVC, respectively.

## 4. Discussion

Pulmonary function among various communities considering genetics, environmental factors, and nutritional status is different (Ahmadial et al., 2006). Comparison between pulmonary function measured in this study and results of other studies from other communities because of difference in anthropometric characteristics of participants is difficult (Bandyopadhyay et al., 2013). Some of the important factors influencing on pulmonary function include: ethnicity, race, gender, age, weight, height (Razi et al., 2005; Ma et al., 2013), physical activity level (Bandyopadhyay et al., 2013), nutrition status (Ahmadial et al., 2006) and environmental factors (Ahmadial et al., 2006) such as air pollution especially air-born particles (Downs et al., 2007; Imboden et al., 2009; Moini et al., 2010; Pala et al., 2012; Bandyopadhyay et al., 2013).

Mean values of respiratory capacities measured in all participants of the current study excluding FEV_1_/VC and FMFT were less than predicted mean values by ECCS reference. Also, mean FVC is equal to the predicted mean value. Mean values of respiratory capacities measured in males and females was the same, except for FVC that was lower in males but it was not significant (p=0.196). FEV_1_/VC values in the current study among males did not show significant relationship with the predicted reference values (p=0.45), but they were significant among women as well as total participants. FVC, FEV1, and FEV1/FVC are pulmonary function parameters which are used in medical estimations for diagnosis of respiratory dysfunction (Downs et al., 2007). In the current study, the measured FVC in participants did not show a significant difference with the reference value, although this value in men was 1.34% less than the predicted value. FEV_1_ and FEV_1_/FC values obtained from all participants showed the significant decrease compared to the predicted values. Spirometry changes showed evidences in support of mild pulmonary lesions, because in pulmonary lesions, FVC value either is normal or it is increased (Neghab et al., 2012). According to the findings, 21.6% of all studied population suffered from obstructive lesions (Table 4). This value among males (24.1%) was more than females (19.6%) and this could be related to more exposure (outdoor jobs) of males with dust storms. Therefore, it could be concluded that exposure to air contaminants such as suspended particles (dust storms) could lead to chronic obstructive lesions (Neghab et al., 2012).

FEV_1_/FVC shows obstructive or restrictive lung disorders (Pal et al., 2010). On the basis of medical evaluations, FEV_1_/FVC less than 70% is considered as asthma (Downs et al., 2007). Various studies have shown that reduction in exposure to airborne particles improves the pulmonary function. Downs et al. 2007 showed that improvement of air quality reduces the annual rate of pulmonary function in adults (Downs et al., 2007). Downs et al. 2007 also reported that improvement of small airways of lung is occurred as a result of the reduction of PM_10_ concentration. They indicated that 109 µg/m^3^ decrease of PM_10_ concentration led to 22% decrease in FEF_25-75%_. This effect was also seen for FEV_1_. Their study was also presented the negative relationship between exposing period to airborne particles and FEV_1_ (Downs et al., 2007).

As seen in Table 1, reduction in FEF_25-75%_ towards FEV_1_ in comparison with the predicted values was significant (p<0.05). Also, values of FEF_25-75%_, FEF_75-85%_, FEF_50%_ and FEF_75%_ showed the most reduction compared to the predicted reference values. Results of spirometry tests showed that in 150 participants, FEF_25-75%_ value was less than 80% and for 24 participants, it was less than 50% of the predicted reference values. In medical examinations, FVC is usually used to evaluate the large airways of lung and FEV_1_ shows the blockage of the small and large airways, while FEF_25%_, FEF_50%_, FEF_75%_, and FEF_25-75%_ are used to show the function of small airways of lung (Pal et al., 2010; He et al., 2010). Albert et al. 1994 showed that FEF_25-75%_ can be applied as a suitable parameter to predict the presence of extreme reactions in the airways (He et al., 2010). Ferguson 1988 also introduced FEF_25-75%_ as a more valuable spirometry factor with respect to PEF to estimate chronic blockage of the airways (He et al., 2010). Considering the above mentioned topics, results of current study could act as a confirmation on previous studies and also as a warning for chronic disorders in small airways due to exposure with dust storms.

VC values obtained in the current study for all participants were not significantly different from VC values reported by Arak and Khomain studies in central Iran (Moini et al., 2010). Arak is considered as an industrial city with high air pollution and Khomain as a non-industrial city with low air pollution (Moini et al., 2010).

FVC value measured in our study for all studied population was not significantly different from FVC value reported by Khomain study (Moini et al., 2010), but more than Arak (Moini et al., 2010) and Kurdistan (Sharifian et al., 2007) studies and less than Sari and Mazandaran studies (Alizadeh et al., 2006; Etemadinezhad and Alizadeh, 2011). Mean FVC value in other studies was ranged from 3 to 3.3 liters which was not significantly different toward values achieved in our research (3.3 lit). Kurdistan province is located in the west of Iran in proximity of Ilam city and it is affected by dust storms similar to Ilam city. Also, Sari and Mazandaran are located in the north of Iran and beside to Caspian lake with wet mild weather and far from air pollution (dust storms).

Mean FEV_1_ calculated in our study was less than results of studies conducted in Mazandaran (Etemadinezhad and Alizadeh, 2011) and Sari (Alizadeh et al., 2006) and it was slightly more than Kurdistan (Sharifian et al. (2007) and Arak (Moini et al., 2010) but in accordance with calculated values in Khomain (Moini et al., 2010). Mean value of FEV_1_ among males was less than of other studies (Alizadeh et al., 2006; Boskabady et al., 2002; Golshan et al., 2003; Razi, et al., 2005; Golshan et al., 2007; Sharifian et al., 2007; Etemadinezhad and Alizadeh, 2011; Li et al., 2012). The same results were also obtained for females, except for Kurdistan study (Sharifian et al., 2007) which our study results were in accordance with the results obtained by Kurdistan study.

Mean value of FEV_1_/FVC measured in this study was less than some studies from Iran (AMRA et al., 2006; Golshan et al., 2007; Alizadeh et al., 2006). Also, mean values of this parameter among all studied population were less than mean values from Sari, Arak and Khomain studies (Alizadeh et al., 2006; Moini et al., 2010). FEV_1_/FVC ratio in our study among females was more than males which was consistent with other studies. Mean PEF values for all studied population in this research were more than values reported by Arak, Khomain and Kurdistan studies (Moini et al., 2010; Sharifian et al., 2007). Mean PEF values in this study were also less than Golshan et al. study in Isfahan (Golshan et al., 2003) and in accordance with Mashhad study (Boskabady et al., 2002). Mean PEF value among females was similar to other studies.

Hong et al. 2010 reported that PEF is considerably reduced among asthmatic patients during dusty days with a high level of PM_10_ particles in South Korea. But, there was no significant relationship between PEF values and PM_10_ and PM_2.5_ concentration among non-asthmatic participants (Hong et al., 2010). Park et al. 2005 reported that PEF was significantly reduced by increasing PM_10_ particles airborne among participants with mild asthma (PARK et al., 2005). Watanabe et al. 2011 also reported that there was a significant correlation between PM_10_ concentration and PEF among patients with worsened symptoms in Asian dusty storms (Watanabe et al., 2011a).

Mean FEF_25-75%_ values in this research for all studied population was less than other studies (Alizadeh et al., 2006; Moini et al., 2010; Etemadinezhad and Alizadeh, 2011; Sharifian et al., 2007). Mean FEF_25%_, FEF_50%_ and FEF_75%_ values calculated in the present study among males and females were less than other studies conducted in Iran (Boskabady et al., 2002; Golshan et al., 2003, 2007) except for mean value of FEF_25%_ which was slightly more than mean value in Isfahan study (Golshan et al., 2007). This difference may be related to the variety of environmental and geographical conditions of different cities in Iran.

Similar to other studies, all pulmonary capacity values showed a negative relationship with age in males except for FMFT that showed a positive correlation with age (Etemadinezhad and Alizadeh, 2011; Rahimian et al., 2001; Ahmadial et al., 2006; Boskabady et al., 2002, Ma et al., 2013; Bandyopadhyay et al., 2013).

Pulmonary function parameters were increased with age increasing during puberty period and then were decreased with age increasing. Golshan et al. 2003 also showed that before age of 20 years, pulmonary function parameters were increased with age and after age of 20 years, these parameters were decreased with age increasing (Golshan et al., 2003). The reason of increase or decrease of pulmonary volumes in various studies may be related to selecting studied populations among different age groups (Bandyopadhyay et al., 2013; Khalilzadeh et al., 2009). Also, like many other studies, all pulmonary capacities in males except for FMFT showed positive significant correlation with height (Bandyopadhyay et al., 2013, Etemadinezhad and Alizadeh, 2011; Ahmadial et al., 2006; Boskabady et al., 2002; Ma et al., 2013; Rahimian et al., 2001). This relationship for females, with less intensity, was the same as males in most pulmonary capacities except for FEV_1_/VC and FEV_1_/FVC parameters which did not show correlation among either males or females. Also, there was not significant correlation between FMFT and height in men and women. This result was presented by other studies (Etemadinezhad and Alizadeh, 2011; Ma et al., 2013; Rahimian et al., 2001).

Pearson’s correlation coefficient showed a positive significant relationship between pulmonary function parameters including VC, FVC, FEF_50%_, FEF_25-75%_ and MVV with weight. This correlation was positive for FEV_1_/FVC, FEF_75%_ and FEF_75-85%_ and it was negative for FEV_1_/VC and FMFT parameters, but all were not significant.

In the current study, there was no significant correlation between BMI and pulmonary function parameters for males. But, VC, FVC, FEV_1_, FEF_75%_, FEF_75-85%_, and MVV showed negative significant correlation with BMI among females (p<0.05). Other respiratory function parameters in females had reversed non-significant correlation with BMI. This may be related to higher BMI in participated females toward males. Ahmadial et al. 2006 presented a negative and non- significant correlation between respiratory parameters and BMI among people with BMI less than 30 kg (Ahmadial et al., 2006). Many other researchers achieved reversed relationship between BMI and respiratory parameters (Bottai et al., 2002). But, Fleg et al. (2001) reported that positive correlation between BMI and respiratory parameters was obtained in youth persons (Harik-Khan et al., 2001). This difference may be due to the muscular strength in youth in comparison with overweight effects in elderly (Harik-Khan et al., 2001).

The results of this research showed a negative significant relationship between duration of inhabitance in Ilam city and all respiratory capacities. Evidences implied that pulmonary functions have been affected by long-term exposure with even relatively low levels of air pollution (Downs et al., 2007). A negative correlation between FEV_1_ and long-term exposure with PM_10_ was also reported in a cross-sectional study in California (Downs et al., 2007). Therefore, reduction of pulmonary functions of Ilamian people could be related to long-term exposure with dust storm in recent decade. The results of this study, Table 5, showed an increase in abnormal respiratory symptoms such as cough and shortness of breath during the dust storm days compared to normal days in either female or males. Also, Fig 1 showed that 68% of the participants during dust storm days felt fatigue and 16% of them suffered from eyes irritations. Fatigue and eye irritation, as the main symptoms of suspended solids in air, have been presented by many studies (He et al., 2010; Li et al., 2012; PARK et al., 2005; Watanabe et al., 2011b). Yamasaki et al. 2011 reported that the number of patients with serious symptoms during Asian dust storms has been significantly increased (Watanabe et al., 2011b). Also, Li et al. 2012 showed that 16-33% of asthmatic patients experienced worsen conditions in their upper and lower respiratory tracts during ADS days (Li et al., 2012). Considering that no study about pulmonary function status in Ilam city, therefore, further studies are needed for confident confirmation of whether the reduction of respiratory capacities among Ilamian people is only related to exposing with dust storms.
